# Carrier Blocking Layer Materials and Application in Organic Photodetectors

**DOI:** 10.3390/nano11061404

**Published:** 2021-05-26

**Authors:** Yi Li, Hu Chen, Jianhua Zhang

**Affiliations:** Key Laboratory of Advanced Display and System Applications of Ministry of Education, Shanghai University, 149 Yanchang Road, Shanghai 200072, China; liyi_shuvip@163.com (Y.L.); chen0305hu@163.com (H.C.)

**Keywords:** organic photodetector, carrier blocking layer, energy alignment, dark current, efficiency

## Abstract

As a promising candidate for next-generation photodetectors, organic photodetectors (OPDs) have gained increasing interest as they offer cost-effective fabrication methods using solution processes and a tunable spectral response range, making them particularly attractive for large area image sensors on lightweight flexible substrates. Carrier blocking layers engineering is very important to the high performance of OPDs that can select a certain charge carriers (holes or electrons) to be collected and suppress another carrier. Carrier blocking layers of OPDs play a critical role in reducing dark current, boosting their efficiency and long-time stability. This Review summarizes various materials for carrier blocking layers and some of the latest progress in OPDs. This provides the reader with guidelines to improve the OPD performance via carrier blocking layers engineering.

## 1. Introduction

### 1.1. Fundamentals of OPDs

Photodetectors can convert optical signals to electronic signals, which are widely applied in optical communication, environmental monitoring, cameras, smart phones, image sensing, and so on [1,2,3,4,5]. Compared to commercial photodetectors such as silicon (Si)- and indium gallium arsenide (InGaAs)-based photodetectors, OPDs are increasingly attractive for light sensing applications as they combine detection wavelength tenability, solution processability, and high photogeneration yield with low fabrication costs, lightweight, and flexibility [6,7]. The basic structure of OPDs generally includes two essentials parts: an active layer for light absorption and electrodes for the collection of charge carriers (electrons and holes). The working mechanism of OPDs is similar to that of organic photovoltaics (OPVs), which can be summarized as (i) the organic semiconductors absorb photons to generate excitons (electron−hole pair) and then the excitons diffuse to donor/acceptor interfaces; (ii) the excitons separate into electrons and holes driven by electric field force formed by extra applied bias; (iii) holes are transferred to the anode through the channels formed by the highest occupied molecular orbital (HOMO) of the donor, and electrons are transported to the cathode via the channels formed by the lowest unoccupied molecular orbital (LUMO) of the acceptor; (iv) the holes and electrons are collected by the corresponding electrode to generate photocurrent.

Continuously improving the high gain property, OPDs have achieved significant breakthroughs and rapid evolution in the last several decades, thanks to the developments of novel donor/acceptor materials, the innovations of the device structure, and interface engineering. Kang et al., reported high-detectivity green-selective all-polymer p-n junction photodetectors by engineering the π-conjugation networks and insulating properties of p- and n-type polymers [8]. Zhang et al., achieved planar heterojunction (PHJ) OPDs based on the medium-band gap fullerene C^60^ and a new low-band gap fused-ring non-fullerene acceptor bilayer structure for a tunable spectral response [9]. Nie reported that aligned nanofibers (ANs) prepared by electrostatic spinning technology as an interfacial layer can significantly enhance the performance of inverted OPDs [10]. Due to the lower relative permittivity (*ε_r_* ≈ 3–4) of organic in comparison with inorganic materials [11], excitons with a relatively high binding energy of ≈ 0.35–0.5 eV are generated after light absorption, rather than free electrons and holes. Thus, the active layer of OPDs is often based on a bulk heterojunction (BHJ) architecture that comprises finely bicontinuous and interpenetrative networks of electron donor and acceptor phases, in which this structure facilitates exciton dissociation and charge transport to the relevant electrodes [12].

### 1.2. Performance Metrics of OPDs

Although figures of merit of OPDs have been summarized in the literature [13,14], here we provide a comprehensive definition of the most important figures of merit to enable a clear understanding of reported OPD performances and key points of attention.

The spectral responsivity (*R*) in units of A W^−1^ describes how much current is generated by the OPD per incoming photon of a given energy. It can be calculated via:

(1) $$R=\frac{J_{light}}{P_{light}}$$

where *J_light_* is the current density under light and *P_light_* is the incident light intensity.

The External Quantum Efficiency (*EQE*) is defined as the ratio of the numbers of collected electrons by the corresponding electrode to the numbers of incoming photons, which can evaluate the OPDs’ capability of converting optical signals into electrical signals, can be expressed as:

(2) $$EQE=R\frac{hv}{q}$$

where *h* is Planck’s constant, v is the frequency of the incident photon, and *q* is the elementary charge. While for the diode type OPD in this review, the *EQE* of OPDs is generally lower than 100% owing to the limited photon harvesting efficiency, exciton dissociation efficiency, charge carrier transport, and collection efficiency [15]. In OPDs, *EQE* (and hence *R*) under reverse bias generally increases with greater external bias voltage due to enhanced charge extraction efficiency but should eventually reach the saturation limit [16,17]. In contrast, the dark current density (*J_d_*) will keep increasing with increasing bias.

The specific detectivity (D*) in units of cm Hz^1/2^ W^−1^ can be expressed as:

(3) $$D^{*}\frac{R\sqrt{AB}}{i_{noise}}=\frac{R\sqrt{A}}{\sqrt{2qI_{dark}}}=\frac{R}{\sqrt{2qJ_{dark}}}$$

where *A* is the area of the device, *B* is the detection bandwidth, *q* is the elementary charge, $i_{noise}$ is the noise current, *I_dark_* is the dark current, and *J_dark_* is the dark current density. Although accurate measurement of $i_{noise}$ is necessary to estimate D*, experimental measurements of $i_{noise}$ are challenging and are not always performed. For the sake of simplicity, the dark current *I_dark_* is generally considered to be the main contribution of $i_{noise}$ [2,18]. Under this assumption, the dark current has a direct effect D*.

The linear dynamic range (*LDR*) is a key parameter to evaluate the capability of the photodetectors to capture changes sufficiently in the light intensity. The *LDR* is defined by the ratio of the maximum to minimum photocurrent and is a vital parameter to evaluate the photodetectors with constant responsivity under different light intensities [19,20]. The LDR in units of dB can be estimated from:

(4) $$LDR=20log\frac{I_{upper}}{I_{lower}}$$

where *I_upper_* and *I_lower_* are the maximum and minimum photocurrent of OPD followed a linear dependence on light intensity under different intensities.

Once electrons and holes are generated by the absorption of photons, they drift towards the relevant electrodes due to the applied electric field [12]. The speed of response, defined as the time required to collect charge carriers at their respective contacts, determines the OPD cut-off frequency and dynamic response. The response speed of photodetectors can be typically expressed as the temporal response, which can be determined by the rise (*t_r_*) and decay (*t_d_*) time of the photo-induced current. The *t_r_* and *t_d_* are defined as the duration time of [21,22], respectively. For BHJ OPDs, it is widely accepted that the response time is limited by the mobility of the slowest holes in the polymer phase [23].

Dark current density (*J_d_*) is defined as the current generated by OPDs under a bias voltage in the absence of light. According to the performance metrics of OPDs described above, the detrimental effects of a high *J_d_* on OPD performance can be evaluated by its negative impact on several key metrics. First, high *J_d_* results in a lower signal to noise ratio and decreases minimum detectable light intensity. Furthermore, the D* decreases for increasing *J_d_* according to Equation (3). Finally, it constrains the *LDR* by increasing the minimum detectable photocurrent according to Equation (4). Exploration of the origins of dark current and solving strategies is essential to develop high-performing OPDs.

### 1.3. Dark Current in OPDs: Origin and Solutions

**Figure 1:**
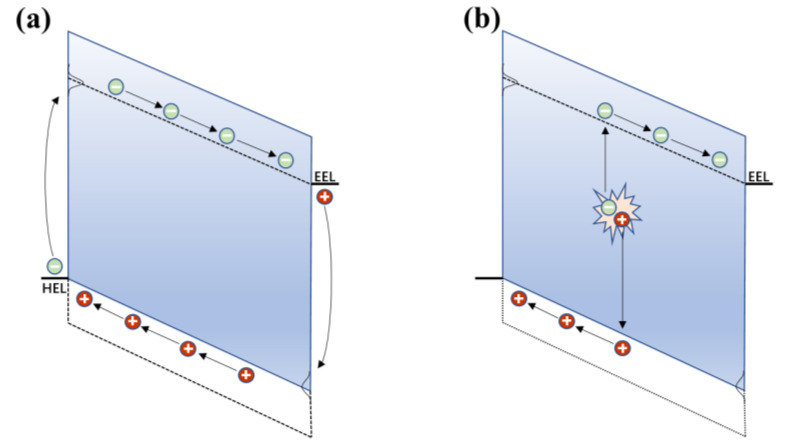
Dark current mechanisms: (**a**) charge injection from the contacts into the corresponding energy level and (**b**) bulk thermal generation of charge carriers within the active layer. (Reproduced with permission from [26]. Royal Society of Chemistry Publishing, 2020).

When the OPD is operated under a reverse bias voltage, intrinsic *J_d_* is mainly attributed to the charge carrier injection rate from the electrode into the semiconductor, or the rate of thermal generation of charge carriers within the active layer, followed by drift towards their respective electrodes under the applied electric field [24,25]. The dark current mechanisms of a typical OPD are shown in Figure 1. The energy levels of the donor and acceptor are represented by the full lines and the dotted lines, respectively.

On the one hand, holes are injected from the low work function electrode into states of the donor HOMO, whereas electrons are injected from the high work function electrode into energy states of the acceptor LUMO. The charge injection rate is expected to be strongly dependent on the energy barrier *E_b_* and applied bias voltage. For electrons, the *E_b_* is defined as the energy difference between the LUMO of the acceptor and the Fermi level of the work function electrode. A higher *E_b_* results in a lower dark current for an applied bias. Assuming perfect Ohmic contacts and alignment between each of the corresponding electrodes’ Fermi level and the acceptor LUMO or donor HOMO, the injection-limited is expected to be proportional to the energy difference between the acceptor LUMO and the donor HOMO. The applied bias increases, resulting in a higher dark current because the electric field causes the charge injection rate to increase. On the other hand, charge carriers are thermally generated and collected by the relevant electrode. It is often said that thermal generation within the bulk organic materials can be neglected due to the large bandgap of organic materials [27].

Based on the above summary of the dark current generation mechanism, the main strategies are summarized to reduce the dark current density in OPDs.

**Figure 2:**
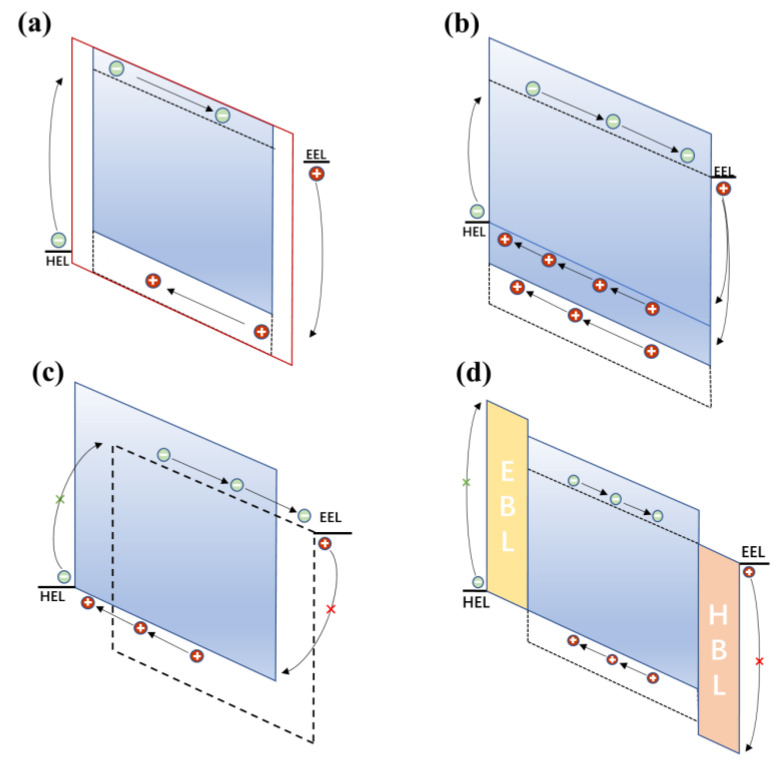
Main strategies to achieve dark current reduction: (**a**) a thick photoactive layer increases the resistance of OPDs; (**b**) a deep HOMO energy of the donor increases the energetic barrier for hole injection; (**c**) vertical phase segregation both reduce charge injection from both electrodes; and (**d**) blocking layers increase the energetic barrier for charge carrier injection.

First, a straightforward way to reduce the dark current of OPDs is to increase the thickness of the photoactive layer (see Figure 2a), due to the increased resistance of BHJ films according to Ohm’s law [28]. However, increasing the thickness of the photoactive layer too much may also negatively affect the photocurrent because of the limited charge carrier mobility of organic materials [29].

Next, *J_d_* can be reduced by choosing an acceptor material with a shallow LUMO to minimize electron injection because of the increased energy barrier *E_b_* of the interface (see Figure 2b). Equally, donor material with a deep HOMO minimizes hole injection. However, the limitations of the synthesis of organic materials in BHJ and the acceptors are mainly fullerene derivatives [4] with similar LUMO in most OPDs.

In addition, another strategy to reduce *J_d_* is to prepare donor and acceptor layer as an interface layer to form pure phases at the related electrodes (see Figure 2c), which can effectively block unfavorable charge carrier injection under reverse bias because pure donor and acceptor materials are also good materials for interfacial layers based on energy level matching. Based on this idea, a PHJ OPD made via sequential deposition of individual donor and acceptor layers has been investigated [30,31,32]. Despite *J_d_* reductions made in device performance upon adopting a PHJ configuration, there was also a drawback associated with this approach. In order to ensure that excitons are able to reach an interface before relaxation, the layers of organic semiconductors are relatively thin, around 20–40 nm, because the excitation diffusion length is between 5 and 10 nm. However, such thin active layers are not able to fully absorb the incident photons reaching a device, limiting the photocurrent and quantum efficiencies that devices can achieve [12].

Another common and effective strategy to reduce *J_d_* by inserting the carrier blocking layer consists of improving charge selectivity at the contacts (see Figure 2d). This is achieved by increasing the energy barrier *E_b_* between the electrode and active layer to suppress charge injection under reverse bias. It also maintains the energy cascade between the active layer and the corresponding electrode to facilitate the extraction of photogenerated carriers. To achieve this, electron blocking layers (EBLs) and hole blocking layers (HBLs) are often employed.

### 1.4. Blocking Layer Engineering

**Figure 3:**
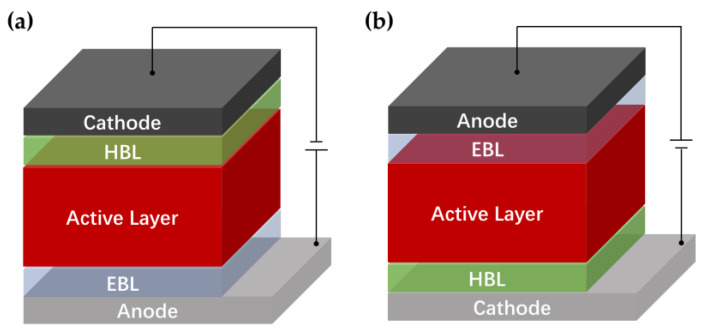
(**a**) Conventional and (**b**) inverted structure of organic photodetectors.

The single-junction OPD is a sandwich structure, which can be divided into a conventional structure (Figure 3a) of the anode/EBL/active layer/HBL/cathode and an inverted structure (Figure 3b) of the anode/EBL/active layer/HBL/cathode [33], shown in Figure 3. In most devices, the bottom electrode ITO can be used as an anode of conventional OPDs or a cathode in inverted OPDs. In the conventional structure, ITO is generally used as an anode for the collection of holes, and in the inverted structure, it is used as a cathode for the collection of electrons. The two-type structure designed is for the better collection of photogeneration carriers.

Generally, an efficient interface layer should meet several requirements in organic devices. First, it can promote the match of energy levels at the interface with decent electrical properties of high electron/hole mobility and the ability to form an ohmic contact with the adjacent active layer and electrode. These comprise an additional interlayer between the electrode and the photoactive layer, such that E_LUMO,HBL_ > E_LUMO,acceptor_ for electrons and E_HOMO,EBL_ < E_HOMO,donor_ for holes, resulting in an increased energetic barrier for charge injection. Ideally, E_LUMO,HBL_ = E_LUMO,acceptor_ and E_HOMO,EBL_ = E_HOMO,donor_ so that carrier collection is not impeded. Second, there is compatibility and stability with the active material and electrode. Finally, relatively high transparency ensures that the active layer can absorb as much light as possible to achieve optimal performance. To date, some reviews on the interface layer of optoelectronic devices have been reported, including organic (e.g., small molecules, polymers, and organometallic complexes) and inorganic materials (e.g., metal salts and metal oxides). 

Considering that these organic devices are generally composed of thin films, their performances are heavily dependent on the interfacial properties, which not only promote the efficient extraction and transport of carriers but also suppress the charge injection under the reverse bias to reduce dark current [26]. Therefore, an ever-increasing number of researchers are working on interface modification, such as HBL and EBL, to improve the performance of OPDs. The interface layers are varied and complex so that it is actually difficult to determine a definite mechanism of one interface material in the device. Some reviews on the interface layer and materials of optoelectronic devices have been reported [34,35,36,37,38,39,40]. Most of them mainly aimed at OPV and focused on some specific interface materials, such as transition metal oxides [34,36], metal oxides [39], two-dimensional materials [38], and conducting polymers [37]. As a device different from OPV, OPDs have unique characteristics in interface engineering. Along with the rapid progress in OPDs, increasingly more interfacial materials have been involved in these devices. In this Review, we aim to provide a summary and discussion on various types of interface (HBLs and EBLs) materials and their latent mechanisms in OPDs. In Section 1, the fundamentals and performance metrics of OPDs will be introduced, and the background and the necessity of blocking layer engineering in OPDs will be presented. Section 2 reviews several carrier blocking layer materials for holes and discusses their modification and performance enhancements in OPDs. Section 3 provides several materials of carrier blocking layer for electrons, resulting in an increased energetic barrier for electrons injection and their applications in OPDs.

## 2. Materials for HBLs in OPD

The HBL needs not only the ability of hole blocking but also the function of electron transport for OPDs, and it mainly includes organic and inorganic materials. It comprises an additional interlayer between the electrode and the organic photoactive layer, such that E_HOMO/CB,HBL_ < E_HOMO,donor_ for holes, resulting in an increased energetic barrier for charge injection [26]. Ideally, E_LUMO,HBL_ = E_LUMO, acceptor_ so that photogenerated carrier collection is not impeded. HBLs based on organic materials have been extensively investigated because of their light weight, solution processability, and large-scale flexible fabrication methods in recent decades. Inorganic HBL materials are favored because of their very good stability, high carrier transport performance, and little absorption losses, which makes the inorganic interface materials widely used in OPDs.

### 2.1. Small Molecular Organic Materials

#### 2.1.1. BCP

**Figure 4:**
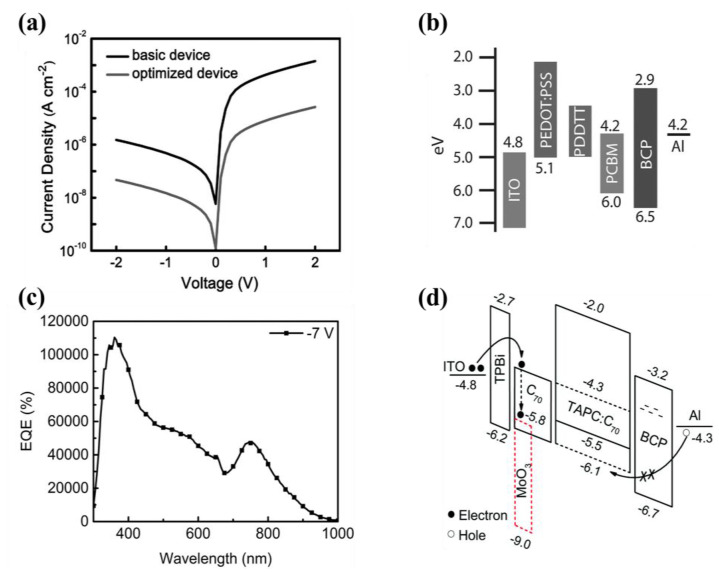
(**a**) Current density-voltage (J–V) characteristics measured in the dark and of the optimized device; (**b**) Schematic energy-level diagrams of the optimized photodetector showing efficient exciton dissociation of the active layer materials and the hole blocking effect of the BCP layer (Reproduced with permission from [44]. WILEY Publishing, 2014). (**c**) Mechanism diagram of the PM-OPDs with the BCP HBLs and MoO_3_ electron trapping layer; (**d**) EQE characteristics of the near-infrared response PMOPDs −7 V bias (Reproduced with permission from [45]. Royal Society of Chemistry Publishing, 2020).

2,9-dimethyl-4,7-diphenylphenanthroline (BCP) is an organic small molecule material, which was used as the functional layer material in organic light-emitting diode (OLED) in the early stage [41]. The thermal evaporated BCP thin film (~10 nm) is commonly used as an interface layer (HBL) due to its low HOMO energy level of ~6.7 eV [42,43]. In Figure 4a, the dark current of the optimized polymer photodetectors decreased significantly by 1–2 orders of magnitude and this device exhibits specific performance with D* of 1.4 × 10^12^ Jones at 800 nm [44]. It is believed that the dark current is dominated by electron injection/transport due to the very large hole barriers from electrode contact or the BCP blocking layer, as shown in Figure 4b. BCP HBL not only has the ability of hole blocking but also has high electron mobility. Guo et al., achieved a higher *EQE* of over 10,000% photomultiplication-type OPDs with the BCP inserted layer, as indicated in Figure 4c [45]. This work shows that HBL materials with larger HOMO, lower LUMO energy levels, and higher electron mobility should be used for the hole accumulation and electron injection in OPD, as shown in Figure 4d. The organic small molecule BCP has also been used in flexible electronic devices. A lightweight ultraviolet (UV) photodetector has been fabricated with mechanical flexibility and photoresponse stability [46]. The photodetector has a low *J_d_* of 1.3 × 10^−5^ mA cm^−2^ even at −15 V bias due to the blocking capability of BCP and the large hole-injection barrier of 1.3 eV from the Al cathode into the HOMO of poly((9,9-dioctylfluorenyl-2,7-diyl)-alt-co-(bithiophene)) (F8T2).

#### 2.1.2. Bphen

**Figure 5:**
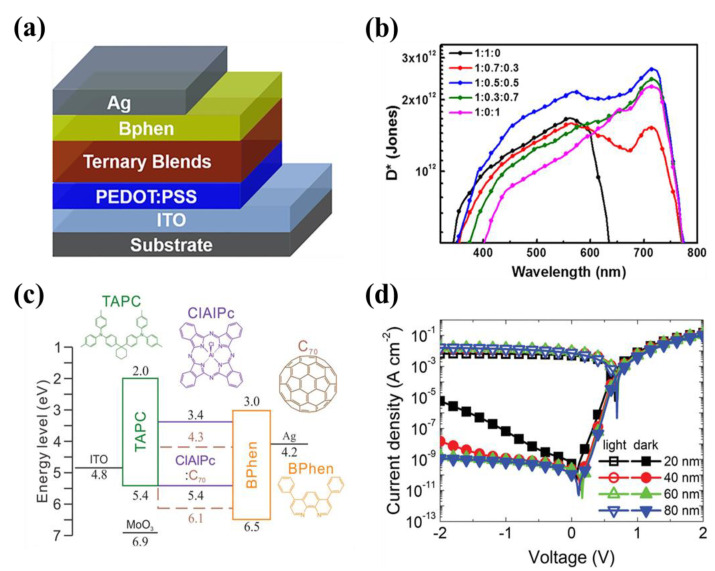
(**a**) Device structure of OPDs with Bphen as the HBL; (**b**) Calculated D* values of OPDs based on various ratios ternary blends system. (Reproduced with permission from [55]. Springer Publishing, 2019). (**c**) The device configuration, molecular structure, and energy level of the respective materials; (**d**) Current density–voltage characteristics (dark and illumination conditions) of OPD with various thicknesses. (Reproduced with permission from [56]. WILEY Publishing, 2020).

4,7-Diphenyl-1,10-phenanthroline (Bphen) is also an organic small molecule material, which was used as the functional layer material in OLED in the early stage [47]. For the interface layer of OPD, Bphen is used to improve charge carrier transport ability due to its electron-transport capability [48]. The HOMO of Bphen is ~6.5 eV, lower than that of universal acceptor materials (e.g., C_60_, PC_60_BM, PC71BM), which can be used as an HBL to reduce dark current under reverse bias in BHJ OPD [49,50,51,52,53]. The optical measurement integration of organic OLEDs and OPDs is implemented with the interface layer of Bphen [3,54]. HBLs of these two optoelectronic devices both use Bphen. Yang et al., demonstrated the broadband visible OPDs with the highest D* reached 2.67 × 10^12^ Jones at 710 nm [55], as shown in Figure 5b. In Figure 5a, Bphen is used as an interface layer to reduce the quenching of photo excitons and impede hole injection from the Ag side. Due to the compatibility between the organic small molecule Bphen and vacuum evaporation process, the evaporated OPD was studied. Lee, et al., fabricated the small molecule OPDs by vacuum-processing with Bphen as HBLs [56], and the device configuration, molecular structure, and energy level of the respective materials are displayed in Figure 5c. The experimental results revealed that the photodetector with the best performance at the wavelength of 730 nm achieved a very low *J_d_* of 1.15 × 10^−9^ A cm^−2^ (Figure 5d) and an *EQE* of 74.6% with a *R* of 0.439 A W^−1^ at −2 V bias.

#### 2.1.3. C_60_

**Figure 6:**
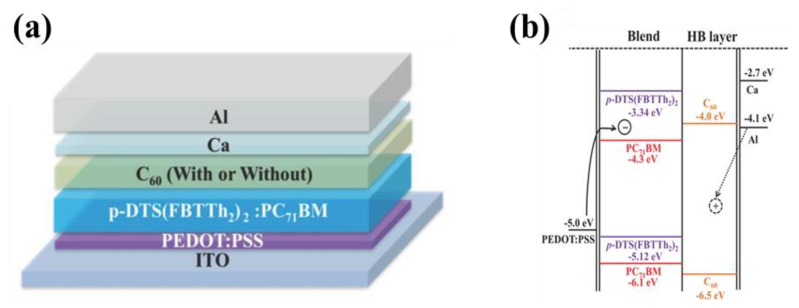
(**a**) Device architecture with and/or without a C_60_ HBL using Ca/Al contact; (**b**) A simplified scheme for the process of dark carrier injection in an Al- and/or Ca/Al-containing photodiode under reverse bias with a C_60_ HBL. (Reproduced with permission from [62]. WILEY Publishing, 2014).

A fullerene, C_60_, has high electron mobility (1.6 cm^2^ V^−1^ s^−1^) and conductivity (2.3 × 10^−3^ S cm^−1^), making it an excellent candidate to extract electrons [57]. Due to the electron mobility and deep HOMO level, the C_60_ layer has been used as an electron transport layer (ETLs) in many organic photoelectric devices [58]. In particular, as HBL in OPDs, C_60_ is a potential candidate expected to reduce dark current [59,60]. Armin and co-authors fabricated a thick junction broadband OPD with a C_60_ (35 nm) HBL to provide hole blocking/electron transport at the interface with the cathode [61]. Kim et al., researched broadband OPDs based on a non-polymeric organic semiconductor in Figure 6a [62]. Hole injection was hindered when a thin layer of C_60_ was added on top of the active layer leading to a lower dark current (0.11 nA cm^−2^) for devices. In Figure 6b, because of the insertion of the C_60_ HBL with a deep HOMO level (~6.0 eV), a low-saturation dark current device even with a thin active layer (350 nm) was realized. Joo and co-authors researched a near-infrared organic thin-film (120 nm) photodiode with 3.3 × 10^12^ Jones D* and 80% *EQE* [63].

### 2.2. Polymer Organic Material

#### 2.2.1. PEIE

**Figure 7:**
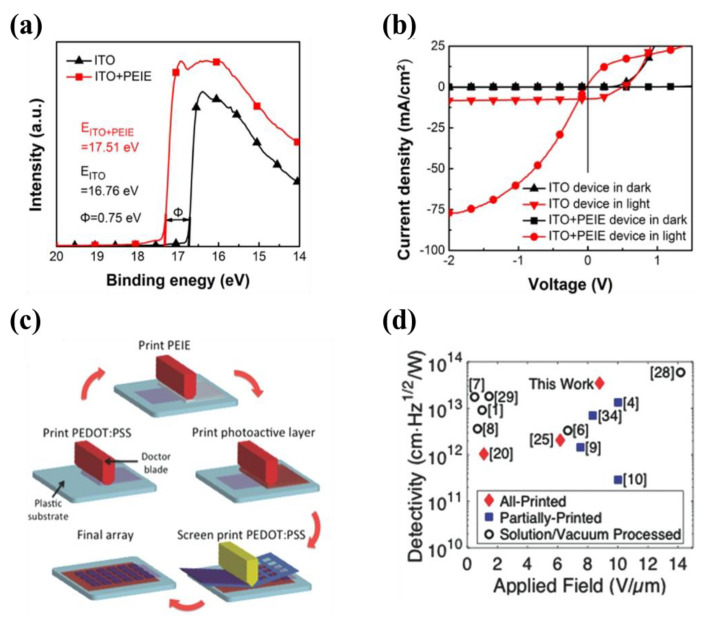
(**a**) UPS spectra of the pristine ITO and PEIE-modified ITO electrodes; (**b**) J-V curves of the OPD on ITO and ITO + PEIE (under AM 1.5 100 mW⋅cm^−2^). (Reproduced with permission from [69]. OSA Publishing, 2017). (**c**) Fabrication process of all-printed organic photodiodes (OPDs); (**d**) Comparison of D* at various applied fields for all-printed OPDs and other OPDs published in the literature. (Reproduced with permission from [70]. WILEY Publishing, 2015).

It is known that Polyethylenimine ethoxylated (PEIE) can shift the work function and lead to an electron selective contact [64] on various different materials, and this polymer material is coated with a one-step, low-temperature solution process. In particular, the low work function transparent PEIE-modified ITO electrode is expected to reduce the dark current in OPDs due to the formed barrier between the active film and the electrode [65,66,67,68]. Wang et al., reported a high-performance solution-processed polymer photodetector with a PEIE-modified ITO electrode [69]. In Figure 7a, the energy barrier formed between the WF of the PEIE-modified ITO and the HOMO of the donor poly(3-hexylthiophene) (P3HT) is 0.75 eV greater than that of the bare ITO-based device. Therefore, the current density is reduced from 2.25 × 10^−5^ A⋅cm^−2^ to 8.79 × 10^−7^ A⋅cm^−2^ at −0.5 V, as presented in Figure 7b. In addition, PEIE can lower the work function of the Poly(3,4-ethylenedioxythiophene)–poly(styrenesulfonate) (PEDOT:PSS) electrode. However, the conventional spin coating process cannot effectively fabricate an adjustable PEIE thin film on PEDOT:PSS due to the solvent orthogonal. The printability of PEIE enables the large-area OPD to be prepared by the solution method [70,71]. Pierre et al. realized all-printed organic photodiodes with the blade-coated PEDOT:PSS/PEIE cathode by changing the weight concentration of PEIE in the blade-coated solution (Figure 7c) [70]. By changing the PEIE solution concentration between 0.05% and 1 wt%, the work function of PEDOT:PSS was able to decrease from 5.15 eV to anywhere between 4.6 and 4.1 eV, and all-printed OPD arrays were obtained with an average D* as high as 3.45 × 10^13^ cm Hz^0.5^ W^−1^ under a bias of −5 V (Figure 7d). Because PEIE has good transmittance in both visible and infrared wavelengths, the PEIE HBL is also used in near-infrared OPDs [72,73].

#### 2.2.2. PFN

**Figure 8:**
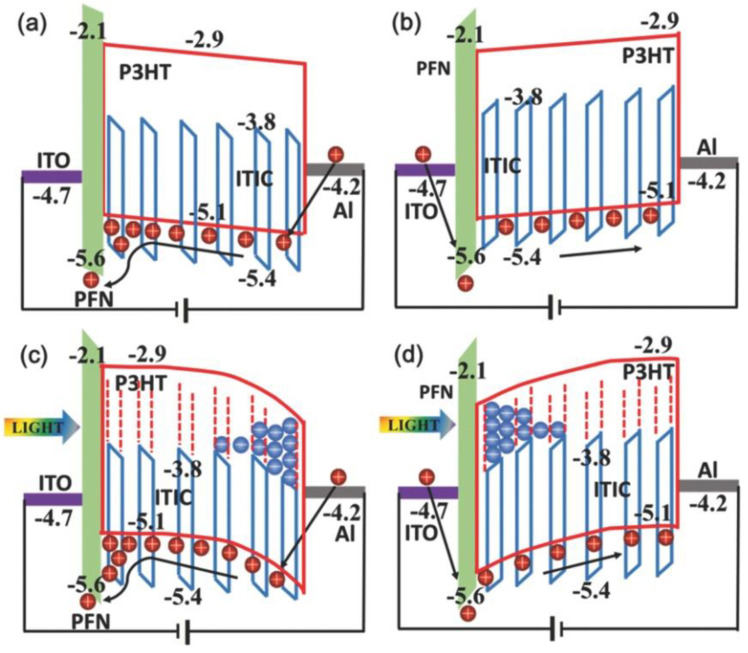
Schematic energy level diagram of the employed materials for the OPDs (**a**,**b**) under dark and (**c**,**d**) light illumination conditions: reverse bias (**a**,**c**), forward bias (**b**,**d**). (Reproduced with permission from [76]. WILEY Publishing, 2016).

The water/alcohol soluble conjugated polymer, poly((9,9-bis(3′-(N,N-dimethylamino)propyl)-2,7-fluorene)-alt-2,7-(9,9-dioctylfluorene)) (PFN) is commonly reported as an interfacial layer for enhancing electron collection in organic solar cells (OSCs) [74]. Some works show that OPDs with PFN as the interfacial layer can work well under forward and reverse bias [75]. Miao et al., used PFN as the anode buffer layer to demonstrate efficient OPDs based on P3HT and non-fullerene [76]. As shown in Figure 8, the value is (about) 0.9 eV between the HOMO levels of ITO and PFN under forward bias and between the HOMO levels of Al and P3HT under reverse bias. In the dark, a large interfacial barrier of 0.9 eV results in hardly hole injections under both forward and reverse bias (Figure 8a,b). With light illumination, trapped electrons in the 3,9-bis(2-methylene-(3-(1,1-dicyanomethylene)-indanone)-5,5,11,11-tetrakis(4-hexylphenyl)-dithieno (2,3-d:2′,3′-d′)-s-indaceno(1,2-b:5,6-b′)dithiophene) (ITIC) near the Al electrode encourage hole tunneling injection and form an external circuit (Figure 8c,d). Wang et al., fabricated the OPDs based on a conventional P3HT/(phenyl-C61-butyric-acid-methyl-ester) (PC61BM) bulk heterojunction by incorporating a PFN interlayer between the anode and the active layer [77]. The *J_d_* was effectively reduced from 0.07 mA cm^−2^ to 1.92 × 10^−5^ mA cm^−2^ under a −0.5 V bias. The holes would pile up close to the PFN and the active layer interface due to the dipole layer of PFN and then create an interfacial band bending leading to a tunneling electron injection to the active layer. Furthermore, Zhong’s team and Xie’s group used a thin layer of PFN-Br as the cathode interlayer layer to facilitate charge collection and prevent the potential diffusion of metal electrodes during evaporation [6,78].

Photomultiplication-type organic photodiodes (PM-OPDs) can be a promising candidate for the commercialization of OPDs based on their self-signal amplification behavior. In BHJ PM-OPDs, it was revealed that Bromide of PFN (PFN–Br) not only reduced the work function of ITO to achieve effective Schottky junctions with polymer donor materials, but also efficiently enhanced the trapping efficiency, which can be ascribed to electrostatic interactions between the positively charged quaternary ammonium groups and trapped electrons within the isolated (6,6)-phenyl C71 butyric acid methyl ester (PC71BM) domains. Zhu’s group prepared the near-infrared and visible light dual-mode OPDs and PFN-Br using a modified ITO anode to create an interface dipole, assisting in bidirectional tunneling hole injection [2].

#### 2.2.3. PEI

The polyelectrolyte material polyethylenimine (PEI) is of great interest for its advantages of low cost, environmentally friendly, and solution-process compatibility. PEI can reduce the work function of electrodes by forming surface dipole moment [79]. Falco et al., sprayed deposition of PEI thin films for the fabrication of fully-sprayed organic photodiodes [80]. In this work, the feasibility of smooth, reliable, and effective spray-coated thin PEI layers was first to demonstrate that exhibit performance comparable to analogous spin-coated films in fully sprayed OPDs. Because the PEI has a good solution processing ability, Grimoldi et al., reported on the successful reduction of dark current below 100 nA cm^−2^ (at −1 V bias) and preserved a high quantum yield (65%) in an inkjet-printed photodetector by the insertion of an electron blocking layer based on PEI [81]. Cesarini et al., fabricated high-performance fully printed organic photodiodes on flexible substrates through the use of a PEI interlayer [82]. Controlling solution composition and deposition parameters for this layer, a 57 nA cm^−2^ *J_d_* was achieved with dramatic improvements in process yield (from less than 20% to more than 90%).

### 2.3. Inorganic Oxide Semiconductor Materials

#### 2.3.1. ZnO

**Figure 9:**
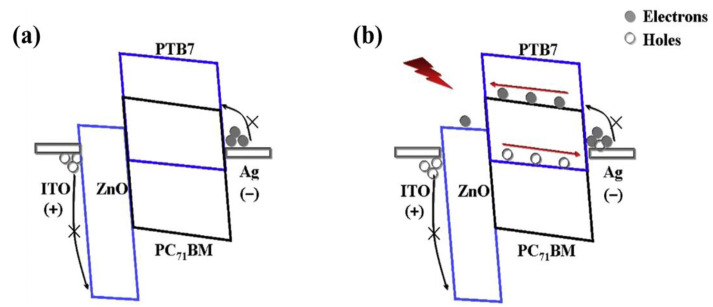
Working mechanism of the OPDs based on PTB7:PC71BM with the ZnO electrode buffer layer under bias: (**a**) in the dark; (**b**) under illumination. (Reproduced with permission from [83]. ELSEVIER Publishing, 2014).

Zinc oxide (ZnO) is the most commonly used ETLs in organic optoelectronic devices. In particular, ZnO layer with a wide band gap can modify both the cathode and anode in OPDs. In inverted OPDs, the ZnO layer is prepared on the bottom electrode, generally between the ITO and the organic layer. Inverted organic optoelectronic devices are more stable and have been extensively studied [33]. For those OPDs, the devices with a ZnO interlayer can usually obtain a lower dark current, an enhanced D*, and an improved *EQE* [83]. As shown in Figure 9, for the OPDs based on poly((4,8-bis((2-ethylhexyl)oxy)benzo(1,2-b:4,5-b’) dithiophene-2,6-diyl)(3-fluoro-2-((2-ethylhexyl)carbonyl)thieno(3,4-b)thiophenediyl)) (PTB7):PC71BM in the dark under reverse bias, the ZnO layer can effectively block the hole injection from ITO into the HOMO of PTB7 due to the large barrier of ~2 eV between ITO and ZnO in Figure 9a, and then the low dark current will be obtained. Under illumination, the photogenerated electrons on the PC71BM LUMO can easily move from the ZnO layer to the ITO electrode under a reverse bias as shown in Figure 9b, which is contributed to obtain the fast photoresponse.

For instance, *n*-type ZnO was selected as the HTL in a semi-tandem structure OPD [84]. The high electron injection barriers enable a *J_d_* as low as 6.51 × 10^−5^ mA cm^−2^ at −0.1 V, resulting in a noise current of 3.91 × 10^−13^ A Hz^−1/2^ at 70 Hz. Since ZnO nanoparticles are stable in the solvent, a print ink was prepared for full-print OPDs. Eckstein et al., proposed a ZnO-nanoparticle-dispersion diluted with butanol in a ratio of 1:2 for use as the electron extraction layer to form a fully digitally printed 2D image sensor [85]. The individual OPD pixels exhibited a state-of-the-art *LDR* (114 dB), *SR* (0.3 A W^−1^), D* (2 × 10^12^ Jones), not only as HBLs, but also to enhance the device response to UV light, because of ZnO absorption in the UV band [17,86,87]. Ma’s group demonstrated a narrow-bandgap OPD had a significant increase in photocurrent upon UV light exposure using ZnO nanoparticles as an anode interfacial layer [88]. Ultrahigh *EQE* of 140,000% was achieved in this device with 30 s UV light irradiation. This phenomenon is attributed to the UV light illumination-induced oxygen molecule desorption from the surface of ZnO nanoparticles, which reduces the electron injection barrier at the anode interface.

ZnO has been widely used as an ETL in OPDs due to its matched work function, high electron mobility, solution processability, and high transparency. However, the size of the sol-gel ZnO nanoparticles is much larger, which may lead to larger interstitial regions and pinholes in the film that negatively affect electron mobility and make the active layer more susceptible to the effects of water and oxygen [89]. By introducing a polymer into the ZnO nanoparticles, a new strategy to prepare ETLs is produced in OPDs [90]. Zhao et al. realized a low dark current and high photo D* transparent organic ultraviolet photodetector by using polymer-modified ZnO as the HBL [87]. The result shows that the aggregation of PFN can cause the surface defects to enhance the possibility of charge carrier trapping, responsible for a lower dark current density. With this combination, a maximum D* of 1.58 × 10^12^ Jones with the fourfold improvement compared with the OPD without PFN has been achieved. The PEIE is also used to modify the ZnO layer in OPDs. Vandewal’s group fabricated infrared OPDs with the ZnO/PEIE HBL, which have the potential to be a useful detector up to 2000 nm [91]. Opoku et al., presented an inverted OPD based on PBDB-T: PC61BM and with a low work function PEIE-modified ZnO on an ITO cathode. This device showed a comparatively broader photoresponse and better performance, with specific D* of 3.749 × 10^12^ and *EQE* of 62% at a bias voltage of 2 V. Xia et al., demonstrated all-polymer and semitransparent OPDs fabricated through lamination on flexible substrates with high D* up to 10^11^ Jones [92]. By introducing the PEI between the active and ZnO layer, this lamination method is roll-to-roll compatible and combined with flexible substrates and is getting close to low-cost, large-scale production.

#### 2.3.2. TiO_2_

Titanium oxide (TiO_2_) is a metal oxide as an efficient ETL because of its high electron mobility, high stability, low-cost, good transparency, and safety for both humans and the environment due to the good match conduction band (~4.4 eV) with the LUMO of PCBM (~4.3 eV) and the deep valence band (~7.5 eV) formed energy barrier Φ_b_ for effectively blocking the holes. By changing the deposition methods and precursor solvents to tune the forming environment, TiO_2_ has four commonly crystal types: anatase (tetragonal), brookite (orthorhombic), rutile (tetragonal), and TiO_2_ (B) (monoclinic). It is widely believed that anatase is preferred over the other crystal types for photoelectronic device applications because of its higher electron mobility and low dielectric constant [93]. Recently, TiO_2_ has been prepared in the form of nanoparticles, nanocrystals, nanotubes, and nanorods [94,95]. The sol–gel method is one of the most widely used conventional methods in the chemical synthesis of TiO_2_ for organic optoelectronic devices [96,97]. This method provides the advantages of homogeneous products and allows the formation of complex shapes. The general preparation procedures are outlined by Jensen et al. [98]. This study addresses the nonaqueous sol–gel synthesis of nanocrystal anatase TiO_2_ and it yields particles 3–7 nm in size.

**Figure 10:**
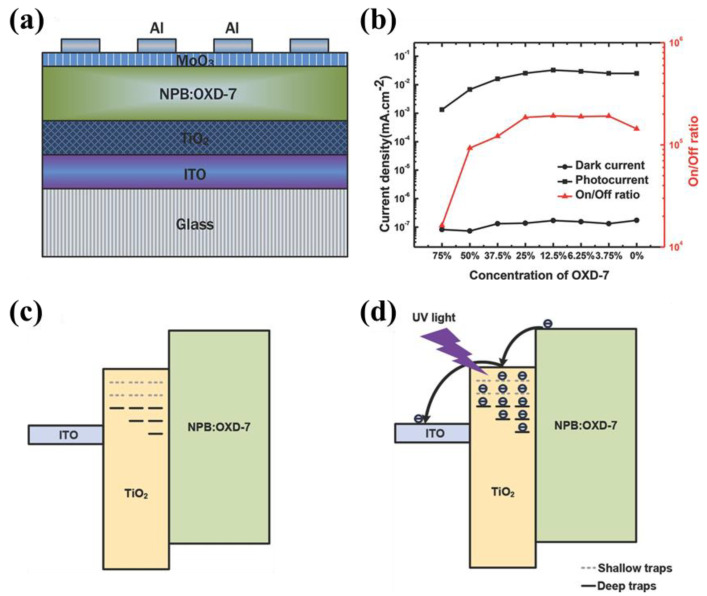
(**a**) Schematic diagram of the device structure of glass/ITO/TiO_2_/NPB:OXD-7/MoO_3_/Al; (**b**)Performance (dark current, photocurrent, and on/off ratio) versus various concentrations of OXD-7 acceptor in the active layer. Schematic diagram showing the working mechanism of the OPDs. (**c**) Under dark room conditions, trapped states in bulk TiO_2_ film induce high impedance contact to block charge injection into the device; (**d**) After UV photo-excitation, the reduced work function and increased conductivity of the TiO_2_ film will induce low-impedance contact at the carrier-extraction layer-metal interface, which facilitates the collection of photogenerated carriers at electrodes. (Reproduced with permission from [99]. WILEY Publishing, 2014).

The early application of TiO_2_ nanocrystals as the electron extraction layer (HBL) in OPDs was reported by Wallace C’s group in Figure 10 [99]. In this case, a clear dark current rectification ratio of approximately 10 at ±1 V is achieved, and the on/off ratio is as high as 10^5^ by incorporating the TiO_2_ (~20 nm), as shown in Figure 10b. In Figure 10c, the effective injection barrier between active and electrode induced by trap states in TiO_2_ will impede charge injection into the device, and thus very low dark current can be obtained [100]. After photogeneration, part of the free electrons are trapped at surface sites and the rest are trapped in the bulk [101]. The shallow bulk trapped electrons relax into deeper bulk sites through a hopping process in Figure 10d. As the occupation increases, the proportion of shallow trapped carriers becomes dominant, resulting in increased mobile charge carriers and their mobility [102,103]. These increased mobile charge carriers will make the Fermi level rise and reduce the work function of TiO_2_, which will lower the effective barriers at the TiO_2_ interfaces. Deng’s group introduced ligand-free anatase TiO_2_ nanocrystals with a clean surface and excellent electron extraction [104]. The grain size of the anatase TiO_2_ nanocrystals is about 4.0 nm, and the surface roughness of the film is about 1.201 nm. By introducing the trap states between the TiO_2_ nanocrystals and the photoactive layer of P3HT:PC61BM, the OPD shows low *J_d_* (3.98 × 10^−7^ A cm^−2^) and high D* (1.9 × 10^12^ Jones) at −1 V.

**Figure 11:**
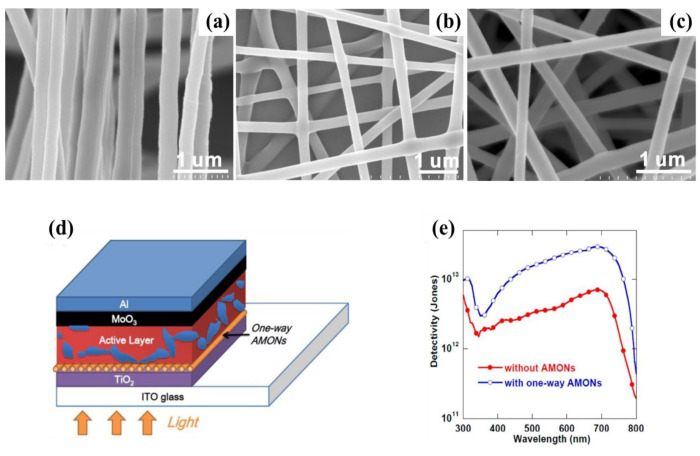
SEM images of (**a**) one-way of TiO_2_, (**b**) multiway of TiO_2_, and (**c**) random of TiO_2_; (**d**) Device architecture of the OPD with one-way of TiO_2_ as the HBL; (**e**) detectivities versus the wavelength under short circuits of OPDs without TiO_2_ or with one-way TiO_2_. (Reproduced with permission from [10]. ACS Publishing, 2014).

In addition, the planar HBL based on nanostructured TiO_2_ has also been introduced in OPDs. By using certain nanostructure materials, the interfacial area between the blocking layer and active layer can be further enlarged. Deng et al., reported aligned nanofibers of TiO_2_ prepared by electrostatic spinning technology as an interfacial layer that can significantly enhance the performance of inverted OPDs [10]. The performance of the devices with TiO_2_ nanofibers in different arrangements (Figure 11a–c) as the interfacial layer was investigated, and the results exhibited that photodetectors with one-way nanofibers had the highest D* of 2.93 × 10^13^ Jones in Figure 11c,d. The enhancement of the performance was attributed to better crystallization of one-way nanofibers of TiO_2_, which facilitate charge separation at the electrode−active interface and electron transport within the interfacial layer.

#### 2.3.3. SnO_2_

**Figure 12:**
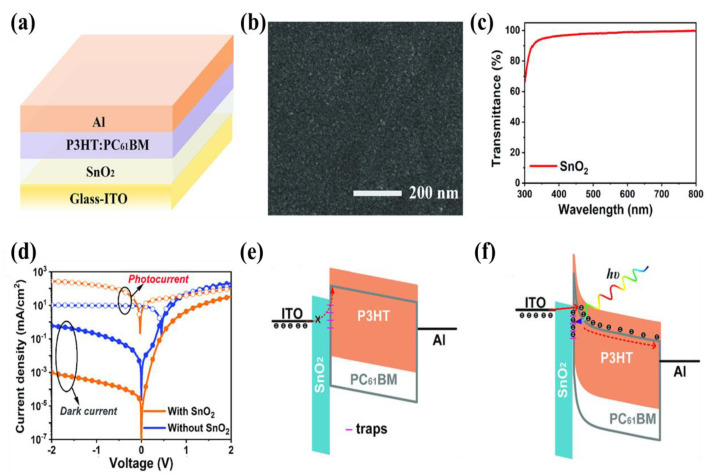
(**a**) Schematic diagram of the photodetector structure; (**b**) SEM image of the non-thermal-and-non-UVO-treated SnO_2_ film; (**c**) Transmittance spectrum of the SnO_2_-coated glass substrates; (**d**) Photocurrent and dark current densities of the devices; The energy band structures of the devices under an applied reverse bias: (**e**) under dark; (**f**) under light. (Reproduced with permission from [107]. WILEY Publishing, 2019).

Stannic oxide (SnO_2_) is another wonderful ETL for photoelectric devices, such as perovskite solar cells, which have a better band alignment with the perovskite layer and high electron mobility of up to 240 cm^2^ V^−1^ s^−1^, which is helpful for electron extraction [105]. Besides, SnO_2_ is easily processed by low-temperature methods (<200 °C), which is compatible with the flexible device and large-scale commercialization [106]. The SnO_2_ layer can efficiently block the external charge injection, which considerably reduces the dark current density in the OPDs. Deng et al., realized a high-performance polymer photodetector using the non-thermal-and-non-ultraviolet–ozone-treated SnO_2_ nanoparticle film between the ITO electrode and the active layer of the P3HT:PCBM blend [107], and the device structure is shown in Figure 12a. In Figure 12b,c the SEM image shows that the untreated SnO_2_ nanoparticle can efficiently form continuous and dense films, and the transmittance spectrum indicates that there is almost no absorption loss when the incident light passes through the non-thermaland-non-UVO-treated SnO_2_ layer into the P3HT:PCBM layer. The *J_d_* of the OPD can be effectively reduced from 1.94 × 10^−1^ to 2.89 × 10^−4^ mA cm^−2^, and the photocurrent density of the device can be significantly increased from 9.63 to 156.63 mA cm^−2^ under −1 V bias, as shown in Figure 12d. According to the working mechanism of the device, the effective injection barrier between the ITO and the untreated SnO_2_ film and the trapped states between the SnO_2_ film and the photoactive layer can significantly impede the charge injection into the device under reverse bias (Figure 12e), leading to a low dark current. The photogenerated charge carriers at the interface between the SnO_2_ and the photoactive layer are trapped due to the defects induced by the non-thermal-and-non-UVO-treated SnO_2_ and the trapped photogenerated electron accumulation at the interface results in a band bending (Figure 12f) [99]. In addition, the conductivity of SnO_2_ increases and the electron injection barrier from ITO to SnO_2_ decreases when the trapped states of the SnO_2_ film are occupied by the charge carriers [88]. Therefore, a large number of electrons can tunnel into the device, leading to a significant photocurrent.

Huang et al., demonstrated an alternative ETL, SnO_2_, which rendered the dark current characteristics minimally sensitive to illumination processes [108]. By using a simple “double”-layer strategy for the SnO_2_ ETL, the magnitude of the dark current can be suppressed to below 10 nA cm^−2^, close to the initial dark current exhibited by the ZnO-based devices without UV exposure history. The original outstanding photoresponse was not compromised with the use of the alternative ETL.

### 2.4. Inorganic Salts

The inorganic salts used as the interfacial materials mainly contain alkali carbonates and alkali metal halides. Alkali carbonates, such as Cs_2_CO_3_, are employed to modify the electrode and blocking carriers in OPDs. Between the electrode and active layer, its appearance can form a dipole moment at the interface, which causes the potential change to increase the holes’ transport energy barrier and to inhibit the recombination of holes by the anode. Guo et al., reported a blue light-sensitive OPD with a thickness of 2 nm Cs_2_CO_3_ HBL [109]. The device showed a *J_d_* of ∼21 nA cm^−2^ at the bias of −3 V, which might be attributed to the carrier injection barrier formed at the interface between the electrode and the sensitive layer.

**Figure 13:**
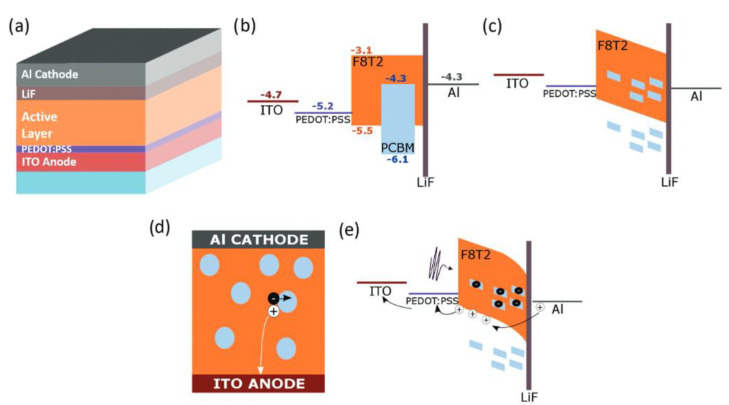
(**a**) Depiction of the device structure: The relative band energies of the layers in the dark (**b**) without and with (**c**) applied reverse bias; (**d**) The active layer morphology with isolated PC71BM clusters and the resulting charge trapping; (**e**) The band energies of the layers under illumination with a reverse bias applied and the resulting photomultiplication mechanism. (Reproduced with permission from [114]. WILEY Publishing, 2017).

The halide salt mainly improves the performance of the photovoltaic device by adjusting the work function of the electrode effectively. Above all, the most common type of these is the LiF, which is widely introduced between the Al electrode and active layer to hinder metal diffusion inside the photoactive matrix [110,111,112]. Zafar et al., presented ternary blend-based bulk heterojunction poly(2,7-(9,9-di-octylfluorene)-alt-4,7-bis(thiophen-2-yl)benzo-2,1,3-thiadiazole) (PFO-DBT): poly(2-methoxy-5(2’-ethylhexyloxy) phenylenevinylene (MEH-PPV):PC71BM organic photodetector [113]. A thin film of LiF (~10 Å) was used between the active layer and top electrode to enhance the performance of the OPD, due to lowering of the effective work function of the top Al cathode and protection of the photoactive layer from hot Al atoms during their evaporated deposition. Esopi et al., fabricated an organic photomultiplier photodetector utilizing F8T2:PC71BM blend active layers with weight ratios of 100:1 and 100:4, with and without the presence of an ETL/HBL of LiF [114]. Generally, devices with LiF are more stable, and reach an *EQE* and R of 5600% and 15.9 A W^−1^, respectively, under 360 nm illumination and a −40 V applied bias and an extremely low dark current of 2.7 × 10^−7^ mA cm^−2^ at a −1 V bias. The device structure and working mechanism are shown in Figure 13. Without or with a reverse bias (Figure 13b,c, respectively), the large hole injection barrier of 1.2 eV from the Al cathode into the HOMO of F8T2 can effectively block hole injection in the dark. Under illumination, these trapped electrons in PC71BM (Figure 13d) near the LiF-modified Al cathode cause the active layer energy bands to bend at the interface with a reverse bias, which lowers the tunneling distance and therefore the barrier for holes to inject from the Al cathode into the HOMO of F8T2 (Figure 13e).

## 3. Materials for EBLs in OPD

The EBLs are in an equal position with HBLs in the OPDs while they serves to extract holes from the organic active layer to the electrode and inhibit the passage of electrons. Similar to the HBLs, some rules should be considered in the design of EBL materials, such that E_LUMO/VB__, EBL_ > E_LUMO, acceptor_ for electrons, resulting in an increased energetic barrier for electron injection. Ideally, E_HOMO/CB,EBL_ = E_HOMO, donor_ so that photogenerated carrier collection is not impeded [26]. There are some EBL materials that mainly include organic and inorganic materials in OPDs.

### 3.1. Small Molecular Organic Materials

#### 3.1.1. TFB

The polymer poly(9,9′-dioctylfluorene-co-N-(4-butylphenyl)diphenylamine) (TFB) is used as an interlayer and it is deposited by a solution-based technique in an organic photoelectric device [115]. Some devices with the interlayer have a significant enhancement due to TFB, which can reduce the WF of the Al electrode. TFB is also deposited on ITO and the PEDOT:PSS electrode to form a barrier against the injection of electrons in OPDs [116,117]. It could provide better energy level alignment with the LUMO of PC71BM and thereby lead to an improvement in the BHJ device containing fullerene derivatives. Keivanidis et al., presented a solution-processed OPD with *J_d_* values as low as 80 pA mm^−2^ and a corresponding *EQE* of 9% [118]. The dark current density of the F8BT:PDI devices is reduced by a factor of ten when a thin TFB EBL acts as a barrier against the injection of electrons from PEDOT:PSS to LUMO_PDI_. Tedde and co-authors reported spray-coated large-area organic photodiodes based on a polymer (Lisicon PV-D4650):PC61BM with the TFB interface layer on the ITO electrode [119]. The values are comparable to commercially available state-of-the-art solid state photodetectors with *J_d_* down to 34 pA cm^−2^ and a maximum *R* of ≈0.44 A W^−1^ (660 nm) under reverse bias conditions of −5 V.

#### 3.1.2. TIPS Pentacene

**Figure 14:**
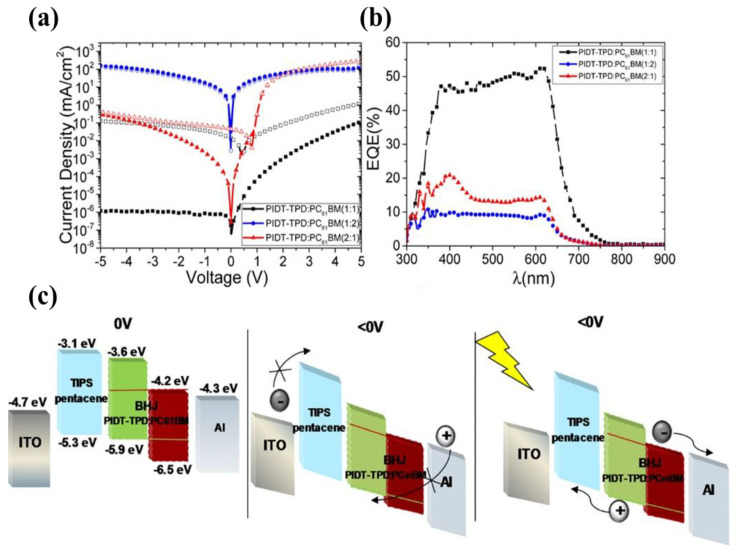
(**a**) J−V characteristics under dark conditions (solid symbols) and under green light at 532 nm at 780 μW cm^−2^ (open symbols) for OPDs comprising different blends ratio; (**b**) *EQE* of the corresponding devices; (**c**) Energy levels and the working mechanism of the OPD in the dark at 0 V, in the dark at reverse bias, and under illumination at reverse bias. (Reproduced with permission from [122]. ACS Publishing, 2018).

Triisopropylsilylethynyl pentacene (TIPS pentacene) is a solution-processed small molecule organic material that is mainly used as an interlayer to enhance the mobility in the fabrication of organic thin-film transistors (OTFTs) [120]. Thanks to its HOMO level of 5.3 eV and LUMO level of 3.1 eV [121], this molecule can be used as an electron blocking interlayer sandwiched between a P3HT: PCBM BHJ and an ITO electrode. In OPDs, the devices with TIPS pentacene EBL present a hysteresis behavior under dark conditions with reverse bias. Tedde et al., reported a conjugated donor−acceptor polymer, poly-(4,4,9,9-tetrakis(4-hexylphenyl)-4,9-dihydro-s-indaceno(1,2-b:5,6-b′)dithiophene-2,7-diyl-alt-5-(2-ethylhexyl)-4H-thieno-(3,4-c)pyrrole-4,6(5H)-dione-1,3-diyl) (PIDT-TPD):PC61BM solution-processed OPDs with the TIPS pentacene interlayer [122]. As shown in Figure 14, the TIPS pentacene interlayer exhibits a high LUMO level (−3.1 eV) and acts as an EBL, which is crucial for reducing the *J_d_* (3 mA cm^−2^ at −5 V bias). The photogenerated carriers are driven by the external negative bias and drift to the respective electrodes under the extraction of the TIPS pentacene layer in the OPD at reverse bias, and the device shows an *EQE* of 52.5% at 610 nm. TIPS pentacene, as a beneficial interlayer for OPDs in imaging applications, has been researched [123]. Benavides et al., reported OPDs with dark currents of∼0.9 nA cm^−2^ at −5 V and *EQE* close to 80% at 530 nm and integrated on top of amorphous silicon thin-film transistor backplanes to obtain the OPD-based image sensor using this interlayer. Compared to the reference, P3HT interlayer, TIPS pentacene shows a significant increase in the OPD’s D* from 3.29 × 10^12^ to 1.63 × 10^13^ Jones.

### 3.2. Polymer Organic Material

#### 3.2.1. PEDOT:PSS

PEDOT:PSS is the conjugated polymer, which is most widely used as EBL to block the transportation of electrons and transport holes in OPDs. It is always composed of poly(3,4-ethylenedioxythiophene) (PEDOT) doped with poly (styrene sulfonate) (PSS), and the PSS is used to improve conductivity and solubility. PEDOT:PSS is widely used in inverted and conventional OPDs because it is strictly solvent orthogonal to the organic BHJ layer. Bouthinon et al., fabricated conventional and inverted OPD with the PEDOT:PSS layer to collect holes [124]. This work brings new elements in the understanding of the impact of oxygen contamination in the performance degradation of organic solar cells or photodiodes. In conventional OPDs, using PEDOT:PSS as the anodic interface to modify ITO could reduce the oxidation potential, enhance conductivity, and is beneficial to the transport of holes and energy level matching [125,126,127]. Zhang’s group [128] reported the ternary OPDs used one interface layer of PEDOT:PSS, which have similar *EQE* values of ≈2000% and specific D* larger than 10^11^ Jones at −50 V bias under the bottom and top illumination conditions. For inverted OPDs, the EBL is deposited on the organic layer and top electrode, and it is required that the preparation of this interface layer does not destructively affect the active layer. Wagner et al. realized semi-transparent inverted organic detectors with a 50 nm PEDOT:PSS layer, which used solution-based fabrication and exhibited an overall transmittance of about 20% in the visible range of the electromagnetic spectrum [129].

**Figure 15:**
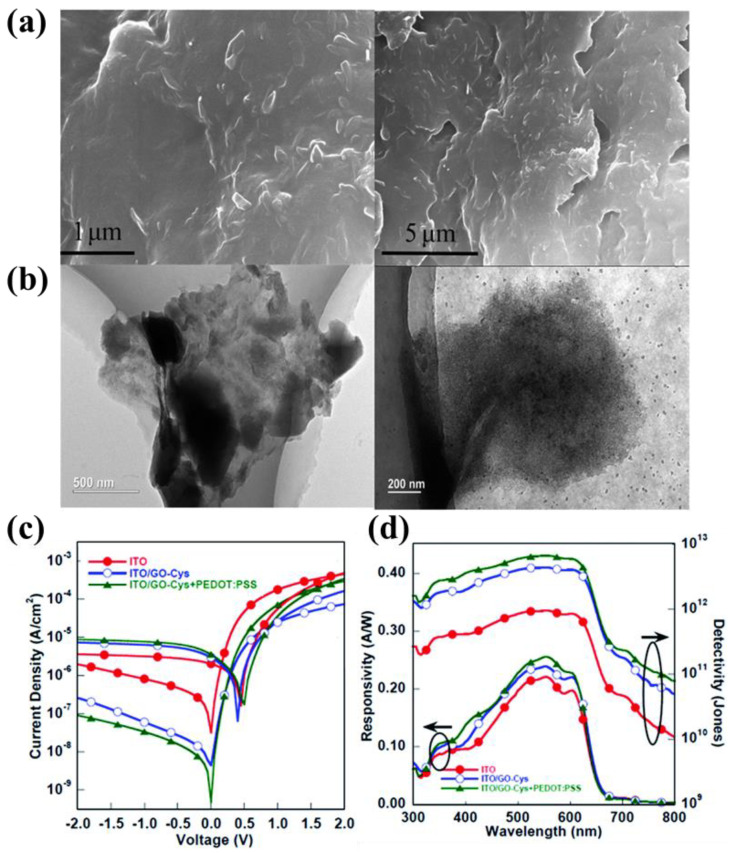
(**a**) SEM image films fabricated with the blend of PEDOT:PSS and GO–Cys; (**b**) TEM images of GO–Cys/PEDOT:PSS composites; (**c**) Current–density to voltage (J–V) characteristics of OPDs with ITO, ITO/GO–Cys or ITO/GO–Cys + PEDOT:PSS as the cathode; (**d**) Responsivity and D* under −0.1 V versus the wavelength of the OPDs. (Reproduced with permission from [130]. The Royal Society of Chemistry Publishing, 2014).

Some groups modified PEDOT:PSS to improve its hole extraction rate and conductivity or change its WF by adding some additives into PEDOT:PSS. P-type inorganic nanoparticles doped into PEDOT:PSS to form a composite interface layer is a potential strategy. Hu’s work [130] demonstrated the work functions of amino acid functionalized graphenes (GO–Cys), and their compounds with PEDOT:PSS were tuned over a wide range, which matched well with the energy of BHJ organic materials. As shown in Figure 15a,b the GO–Cys sheet is tightly covered by the PEODT:PSS, leading to the formation of a contiguous film, and the OPDs exhibited a remarkably low *J_d_* of 4.4 × 10^−10^ A cm^−2^ at 0 V the highest normalized D* of 5.7 × 10^12^ jones at −0.1 V (Figure 15c,d). Abdullah’ group showed that the composite of V_2_O_5_ and PEDOT:PSS was introduced as EBL in the OPD [131]. The resultant device had the ITO/PEDOT:PSS+V_2_O_5_/PCDTBT:PC71BM/V_2_O_5_/Al architecture and exhibited a new range of photo-currents as well as showed an enhanced photo-response.

#### 3.2.2. Donor Materials of BHJ

The BHJ in OPDs consists of donor and acceptor materials, which can effectively promote photon absorption and exciton separation to improve device performance. The donor and acceptor materials not only generate free carriers but also transport carriers under illumination. In particular, the electrons are transported in the high mobility *n*-type layer (acceptor) and holes are transported in the high mobility p-type layer (donor) in PHJ OPDs. The p-type donor material is a potential interfacial EBL in BHJ OPDs [132]. However, the spin-coated donor materials with the BHJ material systems can lower the device performance, because the cross-linking or orthogonal solvent can dissolve or damage the pre-deposited active layer.

**Figure 16:**
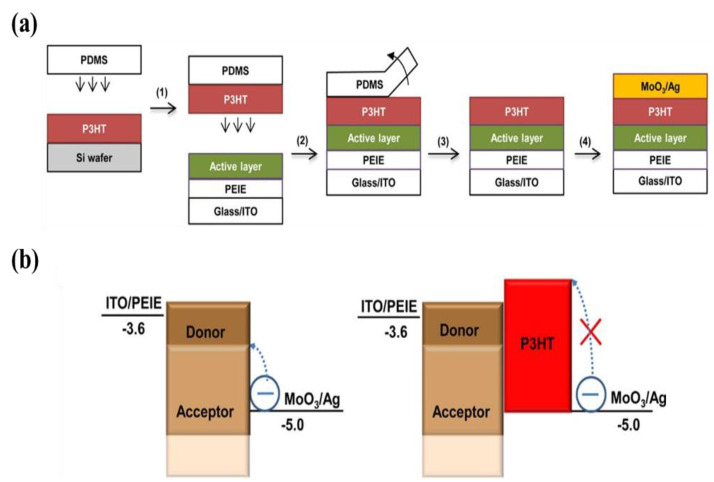
(**a**) Fabrication procedure and device structure of OPDs where a transfer-printed P3HT layer is used as an EBL; (**b**) A schematic demonstrates the P3HT layer blocks the electron injection under reverse bias. (Reproduced with permission from [133]. ACS Publishing, 2014).

A conjugated polymer, P3HT, is an earlier and more common donor material in BHJ organic devices. Xiong et al., introduced a universal strategy of transfer-printing P3HT as the EBL to realize highly sensitive photodetectors [133]. The transfer printing of the P3HT uses poly(dimethylsiloxane) (PDMS) as the transfer medium. The fabrication procedure and a schematic demonstrated the P3HT layer blocks the electron injection under reverse bias, shown in Figure 16. This approach tactfully circumvents the requirement of the solvent orthogonality between the active layer and the P3HT. The insertion of the P3HT EBL substantially reduces the dark current by about three orders of magnitude compared with the photodetectors without the EBL, because it has high-lying LUMO for electron blocking and high hole mobility for hole transport and collection. Inganäs’s group also demonstrated EBLs of P3HT and poly((2,6-(4,8-bis(5-(2-ethylhexyl)thiophen-2-yl)-benzo(1,2-b:4,5-b′)dithiophene))-alt-(5,5-(1′,3′-di-2-thienyl-5′,7′-bis(2-ethylhexyl)benzo(1′,2′-c:4′,5′-c′)dithiophene-4,8-dione)) (PBDB-T) could successfully form on the active layers by using a transfer-printing technique [134]. These inverted all-polymer OPDs exhibit outstanding *EQE* over 70%, low *J_d_* of 1.1 × 10^−8^ A cm^−2^, and high D* over 3.0 × 10^12^ Jones with a planar response over the entire visible range. The aerosol-jet technique is another process to print multilayers from the same solvent system. Hernandez-Sosa and co-authors allowed deposition onto a P3HT-based BHJ without negatively affecting OPD performance [135]. This donor EBL yielded a noise reduction of two orders of magnitude in OPDs operated under −2 V bias.

### 3.3. Inorganic Oxide Semiconductor Materials

P-type semiconductors are used as the HTL materials of optoelectronic devices because of their unique carrier transmission characteristics. p-type metal oxides, such as NiO_x_, MoO_3_, V_2_O_5_, and WO_X_, are competitive materials that currently work as an anode interfacial layer. To some extent, the devices with those metal oxides have similar or better performance compared with those with the most widely used PEDOT: PSS, and the lost-cost synthesis and simpler deposition make them more competitive in mass production.

#### 3.3.1. NiO_x_

Nickel oxide (NiO_x_) has a cubic structure similar to NaCl, with a lattice parameter of 0.4173 nm [136]. The non-stoichiometric NiO_x_ with excess oxygen makes it have a certain number of Ni vacancies, so it will produce holes, making a p-type semiconductor. The large band gap (3.7 eV) of NiO_x_ and interfacial dipole (≥0.6 eV) with the organic active layer leads to a hole-selective interface [137]. For OPD, different device structures (conventional and inverted) use different preparation processes for the NiO_x_ EBL. One method under the vacuum system is that NiO_x_ films were prepared by thermal evaporation of Ni and then annealed at high temperature. Lim et al., reported a conventional OPD using a NiO_x_ anode interlayer to reduce the leakage current [138]. In this work, Ni layers were thermally evaporated in high vacuum at 10^−6^ Torr onto ITO films on glass substrates and then oxidized by heat treatment at 400 °C for 3 h. The D* of the devices is 2.15 × 10^12^ jones, which also have an impressive cut-off frequency of 173.15 kHz at −1 V despite a relatively lower light intensity, which is related to the rapid charge extraction ability of NiO_x_ films.

**Figure 17:**
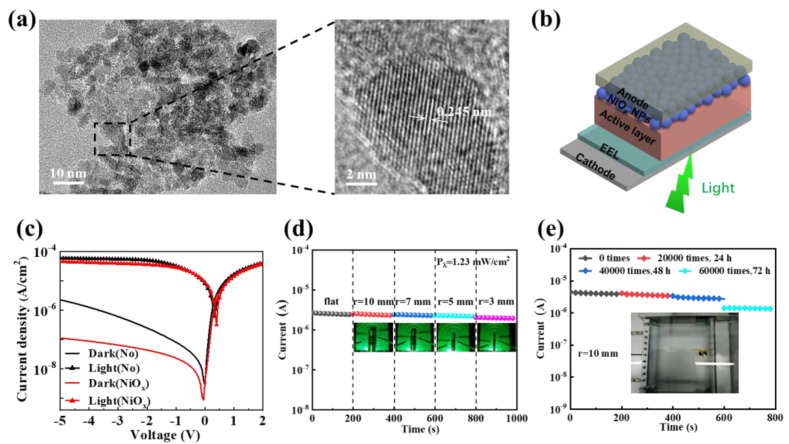
(**a**) TEM image and HRTEM image of NiO_x_ particles; (**b**) Device architecture of inverted OPDs with NiO_x_ as EBL; (**c**) J-V curves of OPD with or without NiO_x_-EBL carried out with 339 µW cm^−2^ green LED light. (Reproduced with permission from [141]. IOPscience Publishing, 2020). Device performance in the photocurrent measured after (**d**) bending at different radii and (**e**) different times of continuous bending. (Reproduced with permission from [142]. IEEE Xplore Publishing, 2020).

Sol–gel methods have been commonly employed to deposit the NiO_x_ films, due to their simple process and good reliability. Manders and co-authors realized low-noise multispectral photodetectors based on all solution-processed inorganic semiconductors with solution-derived NiO_x_ as the EBL [139]. Kim et al., used Ga-doped nickel oxide (NiO_x_) for the EBL and investigated the physical effects of Ga doping on the performance of the organic photodiode [140]. It can be discussed that the leakage current of the OPD with Ga-doped NiO_x_ was significantly decreased owing to the increased LUMO level of this layer. NiO_x_ films can also be deposited through spin-coating with suspensions of pre-synthesized nanoparticles. In this case, NiO_x_ nanoparticles are synthesized and dispersed first, which is a critical step to prepare the functional layer. Li et al., proposed a room-temperature NiO_x_ film synthesized from a NiC_2_O_4_ precursor via hydrothermal treatment and applied it as an EBL to fabricate OPDs [141]. In this work, smaller and more uniform NiO_x_ nanoparticles (5–10 nm) were obtained and demonstrated a high performance OPD, including a *J_d_* of 1.13 × 10^−7^ A cm^−2^, D* of 3.86 × 10^12^ Jones, *R* of 0.74 A W^−1^, and 0.5/8 ms at −5 V (Figure 17). Based on this method, Li and co-authors combined the ultra-small nanoparticles with flexible OPDs [142]. This flexible OPD exhibits outstanding mechanical flexibility following tests with 60,000 bends.

#### 3.3.2. MoO_3_

Molybdenum oxide (MoO_3_) is a typical p-type metal oxide, which has high hole mobility. This metal oxide is been shown to be an interesting alternative to replace PEDOT:PSS as a hole-injection or hole-extraction layer for OLEDs [143], OTFTs [144], and OPVs [145], and OPDs [146]. MoO_3_ has good hydrophilia with the organic layer and is widely used as EBLs in inverted OPDs. In those devices, highly stable electrodes (ITO, Ag) were used, and the interlayer (MoO_3_) was deposited between the active and Ag layer to enhance the stability. Yoon et al., designed a high-performance red-selective thin-film OPD with a dual-band absorbing poly((2,5-bis(2-hexyldecyloxy)phenylene)-alt-(5,6-difluoro-4,7-di(thiophen-2-yl)benzo(c)(1,2,5)-thiadiazole)) (PPDT2FBT) [147]. The MoO_3_ layer was introduced as the EBL and modified the work function of the Ag electrode from −4.2 eV to −5.0 eV, which is beneficial to suppress dark current and enhance the reception of holes. Based on this structure, red-selective OPDs with a high peak D* above 3.0 × 10^12^ Jones with suppressed dark currents (5.9 × 10^−9^ A cm^−2^) were realized. Hwang et al., fabricated an inverted OPD by blending poly(4,8-bis(5-(2-ethylhexyl)thiophen-2-yl)benzo(1,2-b:4,5-b‘)-dithiophene-co-5-(2-hexyldecy1)-1,3-bis(6-octylthieno (3,2-b)thiophen-2-yl)-4H-thieno (3,4-c)pyrrole-4,6(5H)-dione) (PBDTT-8ttTPD) and PC71BM and inserting an 8 nm MoO_3_ EBL. This OPD shows promising photodetecting properties having a low *J_d_* of 3.72 × 10^−9^ A cm^−2^ and high *R* of 0.39 A W^−1^ by varying the thickness of the active layer (260−1100 nm).

**Figure 18:**
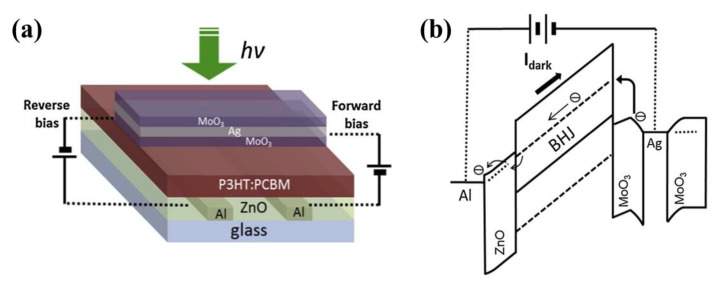
(**a**) The device structure of the top anode OPD with top illumination; (**b**) Band diagram of the OPD with reverse bias under dark conditions. The arrows show the carrier injection mechanism at each electrode and the direction of the dark current. (Reproduced with permission from [148]. ELSEVIER Publishing, 2015).

OPDs have been actively studied as potential low-cost, high-performance alternatives to amorphous silicon (a-Si) photodiode for flat panels. The top illuminated OPDs are necessary to the image array because the a-Si thin-film transistor (TFT) backplane is almost opaque to visible light [5]. The PEDOT:PSS and ultra-thin metallic films can be used as a transparent electrode in these devices, but there are some potential problems, such as the acidity of PEDOT:PSS and the reflection from the metal surface. Kim et al., reported the top anode OPD with MoO_3_/Ag/MoO_3_ semi-transparent electrode for top illumination to yield a high D* of 5.25 × 10^11^ Jones [148]. In Figure 18, the top MoO_3_ layer acts as the passivator to the top electrode, and the bottom MoO_3_ layer not only transmits the incident light but also transports the photogenerated charges. Some image arrays have been realized based on this novel structure. Tessler and co-authors fabricated a hybrid image sensor of small molecule organic photodiode on CMOS [149]. The OPDs used C_70_ as an electron acceptor, a thick TAPC as hole acceptor, and a transparent anode consisting of MoO_3_ (10 nm)/Ag (12 nm)/MoO_3_ (32 nm).

#### 3.3.3. V_2_O_5_

As one of the most important semiconductor nanomaterials, V_2_O_5_ with a work function of ~5.15 eV and a bandgap of ~2.3 eV was demonstrated to be one of the most promising carrier transport materials [150,151]. There are many studies show that V_2_O_5_ deposited by various methods can be used as an alternative HTL in optoelectronic devices [152,153,154]. Gevorgyan’s group fabricated flexible OPD devices with industrial-scale manufacturing techniques such as slot-die coating and demonstrated their use in proximity and light-sensing applications [155]. They studied optimizing the slot-die-coated hydrated, processed using a roll coater and investigated the interfacial properties of V_2_O_5_ with impedance spectroscopy to reveal the loss mechanisms in the electrical properties. Although devices incorporating V_2_O_5_ semiconductor nanomaterials possess better stability in their lifetime, they in general, as a single HTL material, lag behind the PEDOT:PSS HTL because of the non-ideal inorganic nanoparticle film interface. Abdullah [131] combined V_2_O_5_ along with PEDOT:PSS to form an organic-inorganic composite layer and expected that the composite layer would complement the drawbacks of single V_2_O_5_ and conventional PEDOT:PSS. Analysis of the surface roughness over a scan area of 2 μm × 2 μm indicates root mean square (RMS) surface roughness values of 1.27, 2.03, and 4.28 nm for the pristine PEDOT:PSS, composite, and pure V_2_O_5_ layer, respectively. The obtained performance of the device indicates that the incorporation of V_2_O_5_ in PEDOT:PSS to form a composite EBL led to an enhanced photo-response at −1 V, which is desirable for light-sensing applications.

### 3.4. Inorganic Salt

**Figure 19:**
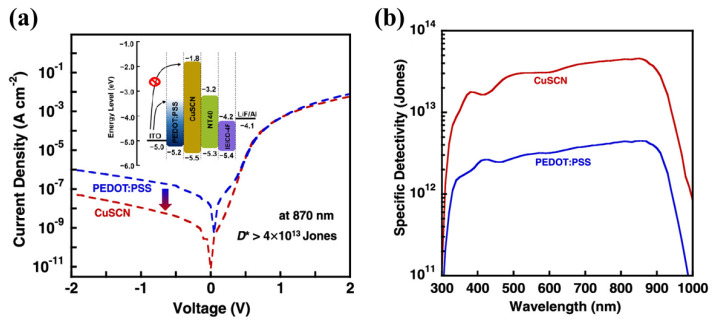
(**a**) Current density versus voltage characteristics of the devices under dark and energy levels of the materials; (**b**) D* curves of photodiodes with CuSCN or PEDOT:PSS interfaces at −0.1 V. (Reproduced with permission from [21]. ACS Publishing, 2021).

At present, the research works on the electron barrier are relatively limited in OPDs. Recent progress indicates that copper(I) thiocyanate (CuSCN) exhibited great potential as the anode interfacial layer due to its good hole mobility, high optical transparency, and remarkable electron blocking ability on account of its relatively shallow conduction band [156,157]. CuSCN has been successfully used in OLEDs and PSCs as HTL, enabling remarkable performance [158]. Huang et al., developed high-performance near-infrared OPDs with the CuSCN interface layer [21]. As shown in Figure 19a, the relatively shallow conduction band of CuSCN resulted in a much higher electron-injection barrier from the anode and shunt resistance compared with PEDOT:PSS, and presented similar optical properties. These features led to the dramatically reduced *J_d_* of 2.7 × 10^−10^ A cm^−2^ and an impressively high specific D* of 4.4 × 10^13^ cm Hz^1/2^ W^−1^ at 870 nm with −0.1 V bias (Figure 19b). Tessler and co-authors reported the dependence of the reverse bias dark current of an acceptor C_70_-based PHJ diode on different donor molecules with a 70 nm solution deposited thick film of CuSCN as the EBL [30]. An inverted small molecule organic photodiode with the structure TiN/CuSCN (80 nm)/TAPC (50 nm)/C_70_ (50 nm)/BCP (8 nm)/Mg (30 nm)/Ag (70 nm) was further fabricated by his group [159].

## 4. Conclusions and Outlook

In this article, we have provided a brief summary of recent progress on different types of blocking layer materials, which have been demonstrated to play a critical role in OPDs. At present, the interface layer materials in organic solar cells and OPDs have great similarities. However, due to the different working mechanisms of devices, not all materials can be used in OPDs. Therefore, more research and optimization of these materials should be carried out for OPDs. From Table 1 and Table 2, we can ascertain the development of OPDs in hole and electron blocking layer materials in recent years, respectively. However, more efforts are needed to promote their applications and to propose new materials for OPDs.

(1) Organic interface materials in OPDs are expected to be used in future flexible wearable electronic devices, and further research is needed. However, it is necessary to develop new strategies to solve the problem that they are orthogonal to the solvent of the organic active layer according to the device structure.
(2) Inorganic materials have also been studied extensively in OPDs because of their high stability. However, the particle size of the material needs to be further reduced, and there are still relatively few p-type inorganic nanomaterials for EBLs. How to achieve the preparation of high-quality inorganic blocking layers at low temperature or even room temperature is the focus of our attention.
(3) Doping is one of the straightforward ways to modify the carrier blocking layer. The method of inorganic nanoparticle doping in organic interface materials is expected to be used in the future research of OPD because it combines the advantages of the two types of materials.

## Figures and Tables

**Table 1:** Survey of the main characteristics of OPD employing materials such as HBLs.

Materials	Device Structure	*J_d_* (A cm^−2^)	*EQE* (%)	D* (Jones)	*R* (A W^−1^)	Measurement Conditions	Ref
BCP	ITO/PEDOT:PSS/active layer/BCP/Al	1.1 × 10^−9^	-	1.4 × 10^12^	0.068	−2 V, @ 800 nm	[44]
	ITO/TPBi/MoO_3_/C70/active layer/BCP/Al	2.2 × 10^−2^	68,927 ^1^	2.2 × 10^12^	188	−6 V, @ 345 nm	[45]
	ITO/PEDOT:PSS/active layer/BCP/Al	1.3 × 10^−5^	2170	8.3 × 10^11^	6.39	−15 V, @ 360 nm	[46]
Bphen	ITO/TAPC:MoO_3_/active layer/Bphen/Ag	1.11 × 10^−9^	41.8	6.43 × 10^12^	0.121	−3 V, @ 360 nm	[51]
	ITO/PEDOT:PSS/active layer/BCP/Ag	4.82 × 10^−4^	-	3.7 × 10^11^	-	−1.5 V, @ 350 nm, 0.5 mW cm^−2^	[52]
	ITO/MoO_3_/CuI/active layer/Bphen/Al	~10^−2^	400	10^12^	-	−8 V, @ 870 nm	[53]
	ITO/PEDOT:PSS/active layer/BCP/Ag	~10^−5^	43.78	2.67 × 10^12^	0.25	@ 710 nm	[55]
	ITO/TAPC/active layer/BCP/Ag	1.15 × 10^−9^	74.6	4.14 × 10^13^	0.439	−2 V, @ 730 nm	[56]
PEIE	ITO/PEIE/active layer/MoO_3_/Ag	7.7 × 10^−9^	60	4.8 × 10^12^	0.24	−1.5 V, @ 546 nm	[65]
	ITO/PEIE/active layer/Al	4 × 10^−5^	12,000	2.27 × 10^12^	54	−0.8 V, @ 530 nm	[66]
	ITO/PEIE/active layer/Au	7.7 × 10^−10^	-	2.2 × 10^13^	0.32	−2 V, @ 629 nm	[67]
	ITO/PEIE/active layer/Al	2.77 × 10^−6^	3200	1.04 × 10^12^	14.25	−1 V, @ 550 nm	[69]
	PEDOT:PSS/PEIE/active layer/PEDOT:PSS	1.5 × 10^−10^	55	3.45 × 10^13^	-	−5 V, @ 530 nm	[70]
PFN	ITO/PFN/active layer/Ag	5.10 × 10^−4^	67.09	2.47 × 10^12^	0.37	−0.5 V, @ 680 nm	[75]
	ITO/PFN/active layer/Al	~10^−3^	650	1.76 × 10^12^	8.7	−15 V, @ 520 nm	[76]
	ITO/PFN/active layer/Al	1.92 × 10^−8^	208.11	9.1 × 10^12^	0.921	−0.5 V, @ 550 nm	[77]
	ITO/PEDOT:PSS/active layer/PFN-Br/Ag	~10^−8^	65	10^13^	-	−10 V, @ 860 nm	[78]
	ITO/PEDOT:PSS/active layer/PFN-Br/Al	4.85 × 10^−10^	56	2.61 × 10^13^	0.33	−0.1 V, 720 nm	[6]
PEI	PEDOT:PSS/PEI/active layer/PEDOT:PSS/PEDOT:PSS	~10^−7^	65	-	-	−4 V	[80]
	PEDOT:PSS/PEI/active layer/Poly-PT/PEDOT:PSS	~10^−7^	65	2.2 × 10^12^	-	−1 V, @ 505 nm	[81]
	PEDOT:PSS/PEI/active layer/PEDOT:PSS	5.7 × 10^−8^	46	3.35 × 10^12^	-	−1 V, @ 525 nm	[82]
C_60_	ITO/PEDOT:PSS/active layer/C_60_/Al	8 × 10^−10^	60	~10^13^	-	−1 V, @ 532 nm	[61]
	ITO/PEDOT:PSS/active layer/C_60_/Al	1.1 × 10^−10^	67.1	9.2 × 10^12^	0.38	−1 V, @ 700 nm	[62]
	ITO/ZnO/active layer/MoO_3_/Ag	6.51 × 10^−8^	~70	2.58 × 10^11^	0.48	−0.1 V, @ 700 nm	[84]
	Ag/ZnO/active layer/PEDOT:PSS	~10^−10^	-	2 × 10^12^	0.3	−1 V, @ 532 nm	[85]
	ITO/ZnO/active layer/MoO_3_/Al	3.9 × 10^−9^	140,000	6.3 × 10^12^	-	−0.5 V, @ 680 nm	[88]
	ITO/ZnO/PEIE/active layer/MoO_3_/Ag	8.075 × 10^−6^	62	3.749 × 10^12^	0.281	2 V, @ 680 nm	[90]
	PEDOT:PSS/ZnO/PEIE/active layer/PEDOT:PSS	2.11 × 10^−8^	~40	~10^11^	~0.17	0 V, @ 700 nm	[92]
TiO_2_	ITO/TiO_2_/active layer/MoO_3_/Ag	~10^−7^	-	~10^12^	0.022	0 V, @ 350 nm	[99]
	ITO/TiO_2_/active layer/Al	1.09 × 10^−7^	113	1.9 × 10^12^	0.5	−1 V, @ 550 nm	[104]
	ITO/TiO_2_/active layer/MoO_3_/Al	~10^−9^	94.22	2.93 × 10^13^	-	0 V, @ 690 nm	[10]
SnO_2_	ITO/SnO_2_/active layer/Al	2.89 × 10^−4^	1430	2.29 × 10^13^	6.97	−1 V, @ 625 nm	[107]
	ITO/SnO_2_/active layer/MoO_3_/Ag	~10^−9^	~70	5.82 × 10^12^	-	−1 V, @ 900 nm	[108]
Cs_2_CO_3_	ITO/PEDOT:PSS/active layer/Cs_2_CO_3_/Al	2.1 × 10^−8^	>100	3 × 10^12^	-	−3 V, @ 450 nm	[109]
LiF	ITO/PEDOT:PSS/active layer/LiF/Al	1.7 × 10^−8^	-	-	0.004	−3 V, @ solar simulator	[113]
	ITO/PEDOT:PSS/active layer/LiF/Al	2.7 × 10^−7^ (−1 V)	5600	-	15.9	−40 V, @ 360 nm	[114]

^1^ Graphene oxide functionalized with cysteine.

**Table 2:** Survey of the main characteristics of OPD-employing materials such as EBLs.

Materials	Device Structure	*J_d_* (A cm^−2^)	*EQE* (%)	D* (Jones)	*R* (A W^−1^)	Measurement Conditions	Ref
TFB	ITO/TFB/active layer/Al	2 × 10^−8^	35	3.34 × 10^12^	-	−5 V, @ 900 nm	[116]
	PEDOT:PSS/TFB/active layer/F8TBT/Al	4 × 10^−9^	20	-	-	−0.5 V, @ 650 nm	[117]
	ITO/PEDOT:PSS/TFB/active layer/Al	8 × 10^−9^	9	-	-	−0.5 V, @ 530 nm	[118]
	ITO/TFB/active layer/Al	3.4 × 10^−11^	82	2.19 × 10^13^	0.44	−5 V, @ 660 nm	[119]
TIPS pentacene	ITO/TIPS pentacene/active layer/Al	1 × 10^−9^	52	1.44 × 10^13^	-	−5 V, @ 610 nm	[122]
	ITO/TIPS pentacene/active layer/Al	9 × 10^−10^	80	1.63 × 10^13^	-	−5 V, @ 530 nm	[123]
PEDOT:PSS	ITO/PEDOT:PSS/active layer/Al	~10^−3^	~250	~6 × 10^11^	0.93	−10 V, @ 400 nm	[126]
	ITO/PEDOT:PSS/active layer/Al	2.68 × 10^−6^	3.92 × 10^5^	1.4 × 10^11^	46.1	−25 V, @ 510 nm	[127]
	ITO/PEDOT:PSS/active layer/Al	~10^−7^	2000	~10^11^	~0.4	−50 V, @ 510 nm	[128]
	ITO/PEDOT:PSS+ GO–Cys ^1^/active layer/Al	~10^−10^	-	5.7 × 10^12^	~0.15	−0.1 V, @ 620 nm	[130]
P3HT	ITO/PEIE/active layer/P3HT/MoO_3_/Al	~1.5 × 10^−9^	-	4.15 × 10^12^	0.19	−0.1 V, @ 525 nm	[133]
	ITO/ZnO/active layer/P3HT/MoO_3_/Al	1.1 × 10^−8^	70	3.0 × 10^12^	0.34	−3 V, @ 630 nm	[134]
	ITO/ZnO/active layer/P3HT/Ag	9 × 10^−5^	-	6.59 × 10^10^	0.214	−2 V, @ 630 nm	[135]
NiO_x_	ITO/NiO_x_/active layer/Yb/Ag	~10^−8^	53.39	2.15 × 10^12^	0.253	−1 V, @ 525 nm	[138]
	ITO/NiO_x_/active layer/ZnO/Ag	3.4 × 10^−8^	~52	1.2 × 10^12^	0.25	−1 V, @ 600 nm	[139]
	ITO/ZnO:Al/active layer/NiO_x_/Ag	1.13 × 10^−7^	-	3.86 × 10^12^	0.74	−5 V, @ 525 nm	[141]
	ITO/ZnO:Al/active layer/NiO_x_/Ag	8.09 × 10^−8^	81.93	2.15 × 10^12^	0.35	−5 V, @ 525 nm	[142]
MoO_3_	ITO/ZnO/active layer/MoO_3_/Ag	5.9 × 10^−9^	-	3.04 × 10^12^	-	−3 V, @ 650 nm	[147]
	Al/ZnO/active layer/MoO_3_/Ag/MoO_3_	2.95 × 10^−7^	31	4.49 × 10^11^	0.14	−1.5 V, @ 650 nm	[148]
	Al/PCBM/active layer/MoO_3_/Ag/MoO_3_	1 × 10^−9^	30	6 × 10^12^	-	−1 V, @ 500 nm	[149]
V_2_O_5_	ITO/PEDOT:PSS + V_2_O_5_/active layer/V_2_O_5_/Al	5.53 × 10^−3^	-	-	-	−1 V, @ (AM 1.5 G) solar simulator	[155]
CuSCN	ITO/CuSCN/active layer/LiF/Al	2.7 × 10^−10^	57.2	4.4 × 10^13^	0.4	−0.1 V, @ 870 nm	[21]
	ITO/CuSCN/active layer/BCP/Mg:Ag	1 × 10^−8^	-	-	-	−1 V	[30]

## Data Availability

No new data were created or analyzed in this study. Data sharing is not applicable to this article.
